# Association of arterial stiffness with a prothrombotic state in uncomplicated nondiabetic hypertensive patients

**DOI:** 10.3389/fcvm.2023.1119516

**Published:** 2023-02-21

**Authors:** Gabriele Brosolo, Andrea Da Porto, Luca Bulfone, Antonio Vacca, Nicole Bertin, Cinzia Vivarelli, Leonardo A. Sechi, Cristiana Catena

**Affiliations:** ^1^Internal Medicine and European Hypertension Excellence Center, Department of Medicine, University of Udine, Udine, Italy; ^2^Diabetes and Metabolism Unit, Department of Medicine, University of Udine, Udine, Italy; ^3^Thrombosis and Hemostasis Unit, Department of Medicine, University of Udine, Udine, Italy

**Keywords:** augmentation index, coagulation, D-dimer, fibrinogen, plasminogen activator-inhibitor 1, pulse-wave velocity

## Abstract

**Background and aims:**

Past studies reported a significant contribution of a prothrombotic state to the development and progression of target organ damage in hypertensive patients. Stiffening of arterial vessels is associated with aging and hypertension, and additional factors could contribute to this process. This study was designed to examine the relationships between arterial stiffening and the hemostatic and fibrinolytic system.

**Methods:**

In 128 middle-aged, nondiabetic, essential hypertensive patients without major cardiovascular and renal complications, we measured coagulation markers that express the spontaneous activation of the hemostatic and fibrinolytic system and assessed stiffness of the arterial tree by measurement of the carotid/femoral pulse wave velocity (cfPWV) and pulse wave analysis with calculation of the brachial augmentation index (AIx).

**Results:**

Levels of fibrinogen (FBG), D-dimer (D-d), and plasminogen activator-inhibitor 1 (PAI-1) were significantly higher in patients with PWV and AIx above the median of the distribution. FBG, D-d, and PAI-1 were significantly and directly related with both cfPWV and AIx, and multivariate regression analysis indicated that the relationships of D-d and PAI-1 with both cfPWV and AIx and of FBG with AIx, were independent of age, body mass index, severity and duration of hypertension, use of antihypertensive drugs, blood glucose, and plasma lipids.

**Conclusion:**

In middle-aged, uncomplicated, nondiabetic patients with essential hypertension, spontaneous activation of plasma hemostatic cascade and impaired fibrinolysis is significantly and independently associated with stiffening of the arterial tree.

## Introduction

Seminal work that was published in the early 1960s described the structural changes of aorta and other elastic vessels in response to blood pressure increase (1). Within the next two decades, assessment of arterial stiffness became relatively common in the clinical setting (2), and arterial stiffness was recognised as a predictor of cardiovascular mortality and morbidity both in the general population (3–5) and in hypertension (6, 7). Currently, arterial stiffness can be estimated by noninvasive and easily accessible methods including measurement of the carotid-femoral pulse wave velocity (cfPWV) and calculation of the augmentation index (AIx) obtained by the pulse wave analisys (PWA), that are accepted tools for evaluation of subclinical vascular damage in hypertension (8). High blood pressure, however, is not the only determinant of arterial changes, and many additional risk factors increase the propensity of hypertensive patients to develop subclinical and clinical vascular abnormalities (9). With regard to arterial stiffness, significant associations with overweight (10), smoking (11), increased plasma lipids levels (12, 13), and impaired glucose tolerance (14) were reported.

Intrinsic hyperactivation of the hemostatic system characterizes the prothrombotic states and is a well recognized risk factor for cardiovascular events in the general population (15, 16) and in patients with hypertension (17, 18) and early renal failure (19). In patients with hypertension, a prothrombotic state has been associated with subclinical changes of the carotid arteries (20, 21), left ventricular diastolic dysfunction (22), and intrarenal hemodynamic changes (23) associated with decreased glomerular filtration rate (24). Some of these adverse effects of the hypercoagulable state in hypertension appear to have a specific link with an activated renin-angiotensin-aldosterone system (25, 26).

The possible relevance of coagulation factors for stiffening of large elastic arteries has been the subject of previous investigations. These investigations were almost exclusively focused on fibrinogen because its circulating levels affect plasma viscosity and, by affecting the rheological properties of blood, might contribute to arterial structural and functional changes (27). Inconsistent results were reported in population studies that examined the relationship of plasma fibrinogen levels with arterial stiffness both in cross-sectional (28, 29) and prospective (30) analyses. Conversely, a positive association of fibrinogen with arterial stiffness was suggested in initial studies that were conducted in healthy subjects (31) and patients with uncomplicated hypertension (32).

It is well known that thorough functional investigation of the hemostatic cascade and fibrinolytic system requires assessment of many other markers in addition to fibrinogen (33), and data on the possible association of these markers with arterial stiffness are completely lacking. For these reasons and for the relevance of arterial stiffening as a predictor of cardiovascular events in hypertension, we examined the relationship between aortic stiffness and extensive markers of hemostatic and fibrinolytic activity in a well characterized group of nondiabetic hypertensive patients who were free of cardiovascular and renal complications.

## Methods

### Patients

One-hundred-twenty-eight consecutive patients with grade 1/grade 2 essential hypertension who presented at the outpatient service of our Clinic from January 2019 to December 2019 were included in a cross-sectional study. Patients seen at the Clinic are white, live in the North-East of Italy, and are representative of the hypertensive population of this regional territory (34). An automatic tool equipped with appropriately sized cuffs (Omron M6, OMRON Healthcare Co., Kyoto, Japan) was used to measure blood pressure in patients who had been supine for at least 15 min, obtaining 3 consecutive readings. According to current guidelines (35), diagnosis of hypertension was done after measurements obtained in at least 3 separate visits. Exclusion criteria were predefined as follows: age younger than 18 or older than 80 years; body mass index (BMI) greater than 35 kg/m^2^; pregnancy; secondary hypertension; 24-h creatinine clearance lower than 30 ml/min/1,73 m^2^; diabetes; use of lipid-lowering, antiplatelet, anticoagulant drugs and any type of drugs that could interfere with the hemostatic system; history of recent illness and acute or chronic inflammatory conditions; history of cerebrovascular, ischemic heart, or peripheral artery disease. Causes of secondary hypertension were ruled out according to current guidelines (35) after clinical, biochemical (urine analysis, creatinine clearance, plasma aldosterone, renin, and cortisol, free urinary cortisol and epinephrine, norepinephrine, and dopamine) and instrumental tests (ECG, echocardiography, renal ultrasound, and renal angio-CT and adrenal CT scan when indicated) (36). Diabetes was excluded by measurement of fasting blood glucose and glycated hemoglobin, and by a standard oral glucose tolerance test (37). Smokers were defined if they smoked for more than 5 years and did not quit more than 1 year before examination. Ethanol consumption (grams/day) was assessed by a standardized questionnaire (38). The leisure physical activity of all patients was estimated by a questionnaire, and patients that practiced at least 3 h of aerobic exercise in a week were defined as physically active. The study was conducted following the statements of the Declaration of Helsinki and was approved by the Institutional Review Board. All patients gave their informed consent.

### Laboratory tests

Venous blood was collected in the early morning after an overnight fast. Plasma was separated and stored at −80°C until processing. Measurements of glucose, total and high-density lipoprotein cholesterol, triglycerides was performed with chemical methods in authomated devices, as previously reported (39). Low-density lipoprotein level was calculated by the Friedewald formula. Glomerular filtration rate was assessed by duplicate measurements of 24-h creatinine clearance. Coagulation parameters were measured in plasma as previously described (40). In brief, fibrinogen was assayed in an automatic coagulometer by a functional test, D-dimer was assayed immunoenzymatically, prothrombin fragment 1 + 2 (F1 + 2) and tissue-plasminogen activator (t-PA) by an enzyme-linked immunosorbent assay, plasminogen activator inhibitor-1 (PAI-1) by immunoassay, antithrombin III (ATIII), protein C, protein S, and von Willebrand factor (vWF) by functional chromogenic assays.

### Arterial stiffness

All measurements were performed in the morning after fasted patients had rested supine in a noiseless room for a minimum of 15 min. Measurements were performed by use of an automated device (AtCor Medical SphygmoCor Xcel Version 1.2.0.7, Sidney, Australia) by the same trained operator who, during examination, was unaware of the patients’ clinical characteristics. Arterial stiffness was assessed by pulse wave analysis with calculation of the AIx and by measurement of the cfPWV.

### Augmentation index

Continuous tracking of the brachial artery pressure waveform was obtained with standard methodology (41), and the average of 2 or more recorded profiles was used for PWA as previously reported (13). The AIx was calculated from the aortic pressure waveform as the difference in height between the first and the second systolic peaks that were expressed as a percentage of the pulse pressure. The intraobserver coefficient of variation of the AIx was 8.4%.

### Pulse wave velocity

Assessment of the cfPWV implies the measurement of the delay between the upstroke of the carotid and femoral artery pulse waves as detected by tonometry (42). To this purpose, the distance between the more readily perceived pulsation of the common carotid artery and the sternal notch and between the sternal notch and the superior edge of the femoral cuff were measured and the sum of the distance carotid-sternal notch + sternal notch-femoral was calculated. The delay between the upstroke of the common carotid and the femoral artery pulse wave was measured and the cfPWV was calculated and expressed in meters/s (m/s) as previously described (13). The intraobserver coefficient of variation of the cfPWV was 3.0%.

### Statistical analysis

The Kolmogorov–Smirnov test was used to determine normality of distribution of the variables included in the study. Normally distributed variables are expressed as mean ± standard deviation and skewed variables as median (interquartile range). Categorical data are expressed as absolute number and percentage. The Student’s *t*-test for unpaired groups and the two-sample Wilcoxon rank-sum test were used for the comparison between 2 groups with normal or skewed variable distribution, respectively. The Pearson’s Chi-square test was used to compare frequency distributions. The relationships between different variables were examined by linear regression analysis, and correlation was expressed by the correlation coefficient *r*. In this analysis, variables with skewed distribution were log transformed. Multivariate regression analysis was performed to determine which variables were independently associated with the AIx and cfPWV. In this analysis, variables were sequentially entered in a stepwise model according to the statistical strenght observed in the univariate analysis. A *p* value of <5% was considered to indicate statistical significance. All data analyses were performed using Stata 12.1 (StataCorp LP, College Station, TX, United States).

## Results

Cross-sectional data of 128 hypertensive patients (age: 52 ± 13 years; 60 men, 68 women) were included in analysis. Forty-seven (37%) of 128 patients had never been treated with anti-hypertensive drugs. The remaining 81patients (63%) were treated with angiotensin-converting enzyme inhibitors or angiotensin II-receptor antagonists (37%), calcium-channel blockers (34%), diuretics (27%), beta-blockers (23%), and alpha-blockers (5%).

Table 1 shows the clinical characteristics and biochemical tests of hypertensive patients who, for statistical reasons, were grouped according to the median value of the AIx (28) and cfPWV (7.6 m/s). Patients with AIx above the median were significantly older and had significantly higher plasma levels of fasting glucose and total and LDL-cholesterol, while no differences were found in sex distribution, BMI, hypertension severity and duration, alcohol intake, frequency of smoking and exercising, and renal function. Patients with cfPWV above the median were also older than those with lower cfPWV and had higher BMI, longer duration of hypertension and higher fasting glucose, but no significant differences in sex distribution, hypertension severity, alcohol intake, frequency of smoking and exercising, renal function, and lipid levels. No significant differences in frequency of use of antihypertensive drugs nor in use of specific antihypertensive drug classes were found between patients with AIx above and below the median value of the distribution (Table 2). Conversely, hypertensive patients with cfPWV above the median were more frequently treated with hypertensive drugs than patients with cfPWV below the median and were more frequent users of renin-angiotensin system blockers and calcium-channel blockers (Table 2).

**Table 1:** Clinical characteristics and biochemical variables of hypertensive patients who were grouped according to the median value of the Augmentation Index (AIx) and carotid-femoral pulse wave velocity (cfPWV).

Variables	All patients (*n* = 128)	AIx ≤median (*n* = 55)	AIx >median (*n* = 73)	*p* value	cfPWV ≤median (*n* = 64)	cfPWV >median (*n* = 64)	*p* value
**Clinical characteristics**							
Age, year	52 ± 13	48 ± 14	54 ± 12	0.012	47 ± 12	57 ± 13	<0.001
Male sex, *n* (%)	60 (47)	29 (53)	31 (42)	0.249	26 (41)	34 (53)	0.156
Body mass index, kg/m^2^	26.7 ± 4.6	26.2 ± 5.2	27.0 ± 4.0	0.302	25.3 ± 4.5	28.0 ± 4.3	0.001
Heart rate, bpm	68 ± 12	68 ± 12	69 ± 12	0.969	68 ± 12	69 ± 12	0.852
Systolic blood pressure, mm Hg	145 ± 19	146 ± 16	144 ± 21	0.604	142 ± 16	148 ± 20	0.081
Diastolic blood pressure, mm Hg	89 ± 12	90 ± 11	89 ± 13	0.432	89 ± 12	89 ± 12	0.874
Duration of hypertension, year	8 ± 9	6 ± 9	9 ± 8	0.074	5 ± 7	10 ± 10	0.001
Smokers, *n* (%)	32 (25)	12 (22)	20 (27)	0.471	18 (28)	14 (22)	0.414
Alcohol intake, g/d	8 ± 12	6 ± 11	9 ± 13	0.251	6 ± 11	9 ± 13	0.166
Physically active, *n* (%)	34 (27)	19 (34)	15 (21)	0.076	19 (30)	15 (23)	0.423
**Biochemical variables**							
Creatinine clearance, ml/min/1.73 m^2^	99 ± 23	97 ± 23	101 ± 21	0.308	97 ± 23	101 ± 21	0.352
Glucose, mg/dL	90 ± 12	86 ± 10	91 ± 13	0.028	87 ± 12	92 ± 12	0.034
Triglycerides, mg/dL	109 ± 58	104 ± 62	114 ± 52	0.372	102 ± 59	115 ± 56	0.247
Total cholesterol, mg/dL	198 ± 46	187 ± 45	210 ± 44	0.009	196 ± 44	199 ± 48	0.738
HDL-cholesterol, mg/dL	58 ± 16	58 ± 16	57 ± 16	0.997	60 ± 17	55 ± 14	0.101
LDL-cholesterol, mg/dL	119 ± 40	110 ± 39	129 ± 38	0.013	118 ± 38	120 ± 42	0.798
**Arterial stiffness variables**							
Augmentation Index	26 ± 12	17 ± 7	38 ± 6	<0.001	24 ± 12	29 ± 11	0.003
Pulse wave velocity, m/s	7.8 ± 1.8	7.3 ± 1.6	8.2 ± 1.8	0.002	6.5 ± 1.0	9.2 ± 1.3	<0.001

Values are expressed as mean ± SD. Median and interquartile range in square brackets are shown for variables with skewed distribution. The Student’s *t*-test for unpaired groups and the two-sample Wilcoxon rank-sum test were used for the comparison between 2 groups with normal or skewed variable distribution, respectively. Reported *p* values for the comparison between patients with an Augmentation Index or carotid-femoral pulse wave velocity above or below the median value of the distribution (respectively: 28 and 7.6 m/s). HDL, high-density lipoprotein; LDL, low-density lipoprotein.

**Table 2:** Use of antihypertensive drugs in the study hypertensive patients who were grouped according to the median value of the Augmentation Index (AIx) and carotid-femoral pulse wave velocity (cfPWV).

Variables	All patients (*n* = 128)	AIx ≤median (*n* = 55)	AIx >median (*n* = 73)	*p* value	cfPWV ≤median (*n* = 64)	cfPWV >median (*n* = 64)	*p* value
Antihypertensive drugs, *n* (%)	81 (63)	30 (54)	51 (70)	0.075	33 (52)	48 (75)	0.006
Renin-angiotensin system blockers, *n* (%)	48 (37)	17 (31)	31 (45)	0.181	18 (28)	30 (47)	0.028
Calcium-channel blockers, *n* (%)	44 (34)	19 (34)	17 (34)	0.972	16 (25)	28 (44)	0.026
Diuretics, *n* (%)	35 (27)	12 (22)	23 (32)	0.223	16 (25)	19 (30)	0.552
Beta blockers, *n* (%)	30 (23)	10 (18)	20 (27)	0.223	12 (19)	18 (28)	0.211
Alpha blockers, *n* (%)	6 (5)	4 (7)	2 (3)	0.230	3 (5)	1 (2)	1.000

Values are expressed as absolute numbers and percentages. The Pearson’s Chi-square test was used to compare frequency distributions. Reported *p* values for the comparison between patients with an Augmentation Index or carotid-femoral pulse wave velocity above or below the median value of the distribution (respectively: 28 and 7.6 m/s).

**Figure 1:**
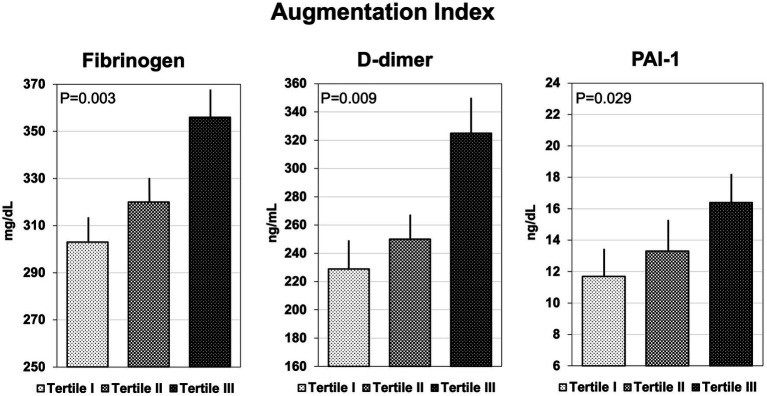
Bar graph showing the plasma concentrations of fibrinogen, D-dimer, and plasminogen-activator inhibitor-1 (PAI-1), across tertiles of Augmentation Index.

**Figure 2:**
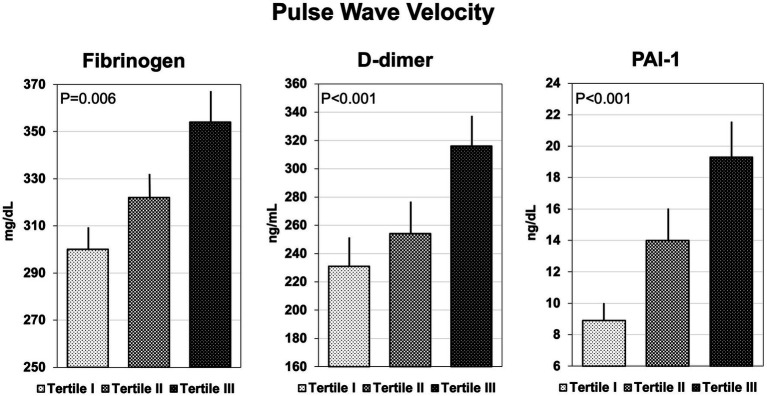
Bar graph showing the plasma concentrations of fibrinogen, D-dimer, and plasminogen-activator inhibitor-1 (PAI-1), across tertiles of pulse wave velocity.

Coagulation parameters are summarized in Table 3. Hypertensive patients with AIx above the median had significantly higher plasma fibrinogen, D-dimer, and PAI-1, but no differences in F1 + 2, t-PA, ATIII, protein C and S, and vWf. Similarly, patients with cfPWV above the median had higher plasma fibrinogen, D-dimer, and PAI-1 than patients with cfPWV below the median, but no significant differences were observed in the remaining hemostatic variables. Plasma levels of fibrinogen, D-dimer, and PAI-1 that were measured in hypertensive patients who were grouped according to tertiles of AIx or cfPWV are shown in Figures 1, 2, respectively. Plasma fibrinogen, D-dimer, and PAI-1 levels increased significantly and progressively across both AIx and cfPWV tertiles.

**Table 3:** Hemostatic variables of hypertensive patients who were grouped according to the median value of the Augmentation Index (AIx) and carotid-femoral pulse wave velocity (cfPWV).

Variables	All patients (*n* = 128)	AIx ≤median (*n* = 55)	AIx >median (*n* = 73)	*p* value	cfPWV ≤median (*n* = 64)	cfPWV >median (*n* = 64)	*p* value
Fibrinogen, mg/dL	322 ± 86	286 ± 59	355 ± 89	<0.001	301 ± 83	342 ± 84	0.013
D-dimer, ng/mL	212 [150–299]	206 [150–254]	225 [162–393]	0.028	206 [150–248]	230 [150–393]	0.010
F1 + 2, pmol/L	175 ± 73	179 ± 70	191 ± 76	0.546	193 ± 68	179 ± 80	0.491
PAI-1, ng/mL	12.1 [7.0–16.6]	9.7 [5.5–15.7]	13.4 [8.7–16.9]	0.006	9.2 [4.4–14.2]	13.9 [9.8–20.5]	<0.001
t-PA, ng/mL	6.4 [4.3–9.0]	6.3 [3.7–9.5]	6.5 [4.7–8.2]	0.855	6.0 [3.7–8.3]	6.8 [5.2–9.6]	0.192
Antithrombin III, %	103 ± 19	100 ± 11	106 ± 27	0.202	102 ± 11	103 ± 26	0.802
Protein C, %	108 ± 17	106 ± 16	110 ± 17	0.179	107 ± 18	108 ± 16	0.730
Protein S, %	94 ± 17	95 ± 17	92 ± 18	0.476	90 ± 17	98 ± 18	0.054
von Willebrand factor, %	131 ± 47	130 ± 50	132 ± 45	0.870	127 ± 47	136 ± 46	0.458

Values are expressed as mean ± SD. Median and interquartile range in square brackets are shown for variables with skewed distribution. The Student’s *t*-test for unpaired groups and the two-sample Wilcoxon rank-sum test were used for the comparison between 2 groups with normal or skewed variable distribution, respectively. Reported *p* values for the comparison between patients with an Augmentation Index or carotid-femoral pulse wave velocity above or below the median value of the distribution (respectively: 28 and 7.6 m/s). F1 + 2, prothrombin fragment 1 + 2; PAI-1, plasminogen activator inhibitor-1; t-PA, tissue-plasminogen activator.

Univariate correlation analysis showed that AIx was significantly and directly related with age, total and LDL-cholesterol, and plasma fibrinogen, D-dimer, and PAI-1 (Table 4). cfPWV was significantly and directly related with age, BMI, systolic blood pressure, duration of hypertension, fasting glucose, and plasma fibrinogen, D-dimer, and PAI-1, and inversely related with HDL cholesterol (Table 4).

**Table 4:** Univariate correlation of the study variables with the Augmentation Index (AIx) and carotid-femoral pulse wave velocity (cfPWV).

Variable	AIx		cfPWV	
	*r*	*p* value	*r*	*p* value
Age	0.308	<0.001	0.442	<0.001
Body mass index	0.125	0.159	0.200	0.024
Heart rate	−0.118	0.188	0.005	0.954
Systolic blood pressure	−0.041	0.647	0.223	0.012
Diastolic blood pressure	−0.071	0.425	−0.061	0.494
Duration of hypertension	0.117	0.190	0.336	0.002
Alcohol intake	0.009	0.923	0.167	0.138
Creatinine clearance	0.044	0.621	−0.122	0.171
Glucose	0.079	0.375	0.345	<0.001
Triglycerides	0.135	0.132	0.094	0.295
Total cholesterol	0.201	0.024	0.037	0.680
HDL-cholesterol	−0.133	0.138	−0.185	0.038
LDL-cholesterol	0.240	0.007	0.098	0.277
Fibrinogen	0.379	<0.001	0.344	<0.001
Log D-dimer	0.210	0.017	0.378	<0.001
F1 + 2	−0.014	0.910	0.053	0.674
Log PAI-1	0.309	0.005	0.352	<0.001
Log t-PA	0.134	0.288	0.163	0.134
Antithrombin III	0.060	0.592	−0.055	0.623
Protein C	0.096	0.389	0.072	0.520
Protein S	−0.163	0.135	0.018	0.870
von Willebrand factor Ag	0.095	0.426	0.136	0.146

Variables with skewed distribution were log transformed for analysis. HDL, high-density lipoprotein; LDL, low-density lipoprotein; F1 + 2, prothrombin fragment 1 + 2; PAI-1, plasminogen activator inhibitor-1; t-PA, tissue-plasminogen activator.

Multivariate regression models were applied using either AIx or cfPWV as the dependent continuous variable and including variables identified in the univariate correlation analysis as covariates. In this analysis, plasma fibrinogen, D-dimer, and PAI-1 were included separately. As shown in Table 5, AIx was directly and independently related with age, LDL-cholesterol and fibrinogen, D-dimer, and PAI-1 plasma levels. cfPWV was directly and independently related with age, body mass index, and plasma D-dimer and PAI-1, and inversely related with HDL-cholesterol (Table 6).

**Table 5:** Multivariate regression models with Augmentation Index (AIx) as the dependent variable and separate inclusion of coagulation variables.

Variable	*β*	*p* value	Variable	*β*	*p* value	Variable	*β*	*p* value
Age	0.261	0.002	Age	0.223	0.013	Age	0.331	0.002
Body mass index	−0.046	0.564	Body mass index	−0.064	0.434	Body mass index	−0.019	0.856
Systolic BP	0.119	0.126	Systolic BP	0.111	0.134	Systolic BP	0.158	0.069
HTN drugs	0.038	0.653	HTN drugs	0.068	0.425	HTN drugs	−0.032	0.769
LDL-cholesterol	0.124	0.116	LDL-cholesterol	0.169	0.032	LDL-cholesterol	−0.041	0.676
Fibrinogen	0.222	0.007	Log D-dimer	0.190	0.027	Log PAI-1	0.239	0.030

Age, body mass index, systolic blood pressure, LDL-cholesterol, and fibrinogen, or log D-dimer, or log PAI-1 were included in analysis as continuous variables. Antihypertensive treatment was included in analysis as a categorical variable (yes/no). BP, blood pressure; HTN, hypertension; LDL, low-density lipoprotein; PAI-1, plasminogen activator-inhibitor 1.

## Discussion

Arterial stiffening anticipates major cardiovascular events in hypertension and many factors, in addition to high blood pressure, can contribute to vascular changes in hypertensive patients. This study was conducted in a highly selected population of hypertensive patients who were free of major cardiovascular and renal complications, testing the hypothesis that a prothrombotic state is associated with arterial stiffening. Results demonstrate that arterial stiffness, as assessed noninvasively by both PWA and cfPWV, is directly related with plasma fibrinogen, D-dimer, and PAI-1 levels. This relationship is independent of age, sex, BMI, hypertension severity and duration, use of antihypertensive drugs, smoking, and plasma lipid levels, suggesting that a prothrombotic state might contribute to functional arterial changes occurring in patients with high blood pressure. Alternatively, in hypertensive patients, stiffening of the arterial tree might activate the hemostatic system leading to a prothrombotic state.

**Table 6:** Multivariate regression models with the carotid-femoral pulse wave velocity (cfPWV) as the dependent variable and separate inclusion of coagulation variables.

Variable	*β*	*p* value	Variable	*β*	*p* value	Variable	*β*	*p* value
Age	0.381	<0.001	Age	0.312	<0.001	Age	0.349	0.001
Body mass index	0.195	0.010	Body mass index	0.158	0.034	Body mass index	0.069	0.451
Systolic BP	0.087	0.291	Systolic BP	0.102	0.247	Systolic BP	0.072	0.299
HTN duration	0.080	0.302	HTN duration	0.076	0.308	HTN duration	0.010	0.921
HTN drugs	0.004	0.957	HTN drugs	0.015	0.839	HTN drugs	−0.032	0.769
Glucose	0.091	0.251	Glucose	0.092	0.233	Glucose	0.150	0.127
HDL-cholesterol	−0.233	0.002	HDL-cholesterol	−0.250	<0.001	HDL-cholesterol	−0.180	0.061
Fibrinogen	0.084	0.247	Log D-dimer	0.224	0.002	Log PAI-1	0.230	0.035

Age, body mass index, systolic blood pressure, hypertension duration, blood glucose, HDL-cholesterol and fibrinogen, or log D-dimer, or log PAI-1 were included in analysis as continuous variables. Antihypertensive treatment was included in analysis as a categorical variable (yes/no). BP, blood pressure; HTN, hypertension; HDL, high-density lipoprotein; PAI-1, plasminogen activator-inhibitor 1.

For a long time, arterial stiffening has been considered just a consequence of aging. This was because, during aging, unrelenting chopping and rearrangement of the elastic layers occurs in the large arterial vessels leading to vascular stiffening (43). Besides aging, however, additional factors were clearly demonstrated to decrease elasticity of the arterial wall, including smoking (11), obesity (10), dyslipidemia (12, 13), diabetes (14, 44) and, last but not least, hypertension (8). Chronic blood pressure elevation is in fact associated with structural abnormalities of the vascular wall with a geometric remodeling that results from thickening of the muscular layer and increase in extracellular matrix (45). These structural changes result in functional abnormalities of the elastic arteries that are associated with an increased risk of hypertension-related cardiovascular complications (46).

Non-invasive methods are widely employed for definition of arterial stiffness. In the last decades, assessment of the cfPWV has become extensively popular and because of its capacity to define the distensibility of large conduit arteries it was considered a sort of gold standard. Additional methods that are based on recording and analysis of the blood pressure waveform such as the AIx explore the distensibility of the peripheral arterial tree (47) and are complementary to measurement of cfPWV. In this study, both cfPWV and AIx were measured and were found to be independently related with levels of hemostatic and fibrinolytic variables that suggest the existence of a prothrombotic state.

Increased intrinsic activity of the hemostatic cascade and impaired fibrinolysis were reported in patients with hypertension (48, 49) and result in a higher risk of thrombotic events. In these patients, a prothrombotic state was in fact associated with major cardiac and vascular events (17), but also with subclinical structural changes of the heart (22) and carotid arteries (20). The independent association of higher levels of fibrinogen, D-dimer, and PAI-1 with stiffening of the large conduit arteries and peripheral arterial tree reported in this study supports the contribution of a prothrombotic state also to hypertension-related functional changes of the entire vascular system. In addition to its role in multiple steps of the coagulation process, fibrinogen increases plasma viscosity and the shear stress of the vascular wall causing rheological changes in the systemic and local circulation (50). Moreover, fibrinogen promotes proliferation of vascular smooth muscle cells and fibroblast migration to the vascular wall (51), possibly leading to decreased arterial distensibility. D-dimer is the major product of fibrin breakdown and a reliable biochemical marker of the overall state of activation of the coagulation system. Higher plasma D-dimer levels together with increased plasma fibrinogen indicate the existence of a prothrombotic state that might adversely affect endothelial function with decreased generation of nitric oxide possibly contributing to arterial stiffening (52). On the other hand, independent association of cfPWV and AIx with higher PAI-1 levels suggest also a contribution of an impaired fibrinolytic system to the prothombotic state that is associated with arterial stiffening.

The relationship between coagulation variables and arterial stiffness was investigated in previous studies. Early observations obtained in healthy middle-aged women did not report any association between PWV and plasma fibrinogen (53). Higher concentrations of fibrinogen and D-dimer were subsequently reported in association with increased AIx in healthy volunteers (31), whereas another study observed an association of PAI-1 but not D-dimer with PWV (54). In a cross-sectional analysis of the Rotterdam Study, no significant associations were found between plasma fibrinogen concentration and aortic and carotid stiffness (28), and in 2,000 participants of the Framingham Offspring Study neither fibrinogen nor PAI-1 were associated with either cfPWV or AIx (29, 55). In the Northern Ireland Young Heart Project, however, the association between the metabolic syndrome and aortic-iliac PWV was found to be mediated, among other factors, by plasma fibrinogen levels (56), and in the Caerphilly prospective study plasma fibrinogen concentrations predicted higher AIx values after a 20-year follow-up. In hypertensive patients, Vlachopoulos et al. reported that increased plasma fibrinogen was independently associated with the cfPWV but not AIx (32), suggesting a different contribution to functional changes of large elastic and more peripheral arteries. However, no relationship of plasma fibrinogen with AIx (57) and cfPWV (58) was found in two other studies conducted in patients with hypertension. Thus, results obtained previously in healthy subjects, in the general population, and in hypertensive patients are highly controversial as to the relationship between coagulation and arterial stiffness. The major strength of our study was that we examined a broad panel of hemostatic variables in a highly selected group of relatively young hypertensive patients, reporting a significant and consistent independent association between a prothrombotic state and both cfPWV and AIx.

Limitations to the present study should also be considered. The cross-sectional design limits the possibility to establish a causal relationship between the prothrombotic state and arterial stiffness or viceversa, although independence of this relationship from confounders in the multivariate analysis would suggest so. Also, the possibility that a prothrombotic state and arterial stiffening causally affect one another cannot be excluded. Use of a selected clinic sample of hypertensive patients might limit the possibility to extend the present findings to a broader context. Inclusion of a significant proportion of patients who were treated with antihypertensive drugs might have affected the results although possible interference of treatments on the results were ruled out in the multivariate analysis. Last, calculation of the pulse wave travel distance as the sum of the distance carotid-sternal notch plus the distance sternal notch-femoral pulsation might have overestimated the aortic path length as compared to other suggested methods for PWV assessment (59).

## Conclusion

Identification of conditions that in addition to high blood pressure could contribute to the development of subclinical arterial damage is crucial for effective prevention of major complications in patients with hypertension. Arterial stiffening is an unrelenting process related to aging that is accelerated in patients with high blood pressure and predicts cardiovascular morbidity and mortality. Because of previous evidence on a role of a prothrombotic state in the development and progression of hypertension-related organ damage, we examined the relationship between arterial stiffness and variables of the hemostatic and fibrinolytic systems. This is the first study to report an independent association of arterial stiffening with activated hemostatic cascade and impaired fibrinolytic system in nondiabetic hypertensive patients who were free of major organ complications. These results could have important clinical implications opening new paths to the possibility to prevent hypertensive vascular changes. Detection of changes in the hemostatic system could be useful to guide physicians toward more aggressive control of blood pressure and additional risk factors. Future studies will have to test the effects of interventions on the hemostatic system on the development and progression of hypertension-related vascular abnormalities.

## Data availability statement

The raw data supporting the conclusions of this article will be made available by the authors, without undue reservation.

## Ethics statement

The studies involving human participants were reviewed and approved by Institutional Review Board - Department of Medicine. The patients/participants provided their written informed consent to participate in this study.

## Author contributions

GB, LS, and CC: conceptualization and design. GB, AP, LB, AV, NB, CV, LS, and CC: acquisition of data and data analysis. GB, LS, and CC: writing the original draft. AP, LB, AV, NB, and CV: writing, reviewing, and editing. All authors approve the final version of the manuscript, including the authorship list and agree to be accountable for all aspects of the work in ensuring that questions related to the accuracy or integrity of any part of the work are appropriately investigated and resolved.

## Funding

This work was supported by a research grant of the PierSilverio Nassimbeni Foundation to LS and CC.

## Conflict of interest

The authors declare that the research was conducted in the absence of any commercial or financial relationships that could be construed as a potential conflict of interest.

## Publisher’s note

All claims expressed in this article are solely those of the authors and do not necessarily represent those of their affiliated organizations, or those of the publisher, the editors and the reviewers. Any product that may be evaluated in this article, or claim that may be made by its manufacturer, is not guaranteed or endorsed by the publisher.

## References

[1] Wolinsky H Glagov S. Structural basis for the static mechanical properties of the aortic media. Circ Res. (1964) 14:400–13. doi: 10.1161/01.RES.14.5.400, PMID: 14156860

[2] Safar ME Levy BI Struijker-Boudier H. Current perspectives on arterial stiffness and pulse pressure in hypertension and cardiovascular diseases. Circulation. (2003) 107:2864–9. doi: 10.1161/01.CIR.0000069826.36125.B4, PMID: 12796414

[3] Shokawa T Imazu M Yamamoto H Toyofuku M Tasaki N Okimoto T. Pulse wave velocity predicts cardiovascular mortality: findings from the Hawaii-Los Angeles-Hiroshima study. Circ J. (2005) 69:259–64. doi: 10.1253/circj.69.259, PMID: 15731528

[4] Mattace-Raso FU van der Cammen TJ Hofman A van Popele NM Bos ML Schalekamp MA. Arterial stiffness and risk of coronary heart disease and stroke. The rotterdam study. Circulation. (2006) 113:657–63. doi: 10.1161/CIRCULATIONAHA.105.555235 16461838

[5] Willum Hansen T Staessen JA Torp-Pedersen C Rasmussen S Thijs L Ibsen H. Prognostic value of aortic pulse wave velocity as index of arterial stiffness in the general population. Circulation. (2006) 113:664–70. doi: 10.1161/CIRCULATIONAHA.105.579342, PMID: 16461839

[6] Laurent S Boutouyrie P Asmar R Gautier I Laloux B Guize L. Aortic stiffness is an independent predictor of all-cause and cardiovascular mortality in hypertensive patients. Hypertension. (2001) 37:1236–41. doi: 10.1161/01.HYP.37.5.1236 11358934

[7] Laurent S Katsahian S Fassot C Tropeano AI Gautier I Laloux B. Aortic stiffness is an independent predictor of fatal stroke in essential hypertension. Stroke. (2003) 34:1203–6. doi: 10.1161/01.STR.0000065428.03209.64, PMID: 12677025

[8] Safar ME Asmar R Benetos A Blacher J Boutouyrie P Lacolley P. Interaction between hypertension and arterial stiffness. An expert reappraisal. Hypertension. (2018) 72:796–805. doi: 10.1161/HYPERTENSIONAHA.118.11212 30354723

[9] Brosolo G Da Porto A Catena C Sechi LA. Arterial stiffening in hypertension: is it just high blood pressure? Rev Cardiovasc Med. (2021) 22:1073–5. doi: 10.31083/j.rcm2204117 34957752

[10] Wildman RP Farhat GN Patel AS Mackey RH Brockwell S Thompson T. Weight change is associated with change in arterial stiffness among healthy young adults. Hypertension. (2005) 45:187–92. doi: 10.1161/01.HYP.0000152200.10578.5d, PMID: 15596570

[11] Jatoi NA Jerrard-Dunne P Feely J Mahmud A. Impact of smoking and smoking cessation on arterial stiffness and aortic wave reflection in hypertension. Hypertension. (2007) 49:981–5. doi: 10.1161/HYPERTENSIONAHA.107.087338, PMID: 17372029

[12] Wilkinson IB Prasad K Hall IR Thomas A MacCallum H Webb DJ. Increased central pulse pressure and augmentation index in subjects with hypercholesterolemia. J Am Coll Cardiol. (2002) 39:1005–11. doi: 10.1016/S0735-1097(02)01723-0, PMID: 11897443

[13] Brosolo G da Porto A Bulfone L Vacca A Bertin N Colussi GL. Plasma lipoprotein(a) levels as determinant of arterial stiffening in hypertension. Biomedicine. (2021) 9:1510. doi: 10.3390/biomedicines9111510, PMID: 34829739 PMC8615029

[14] Catena C Colussi GL Frangipane A Russo A Verheyen ND Sechi LA. Carotid artery stiffness is related to hyperinsulinemia and insulin-resistance in middle-aged, nondiabetic hypertensive patients. Nutr Metab Cardiovasc Dis. (2015) 25:968–74. doi: 10.1016/j.numecd.2015.06.009, PMID: 26234565

[15] Ridker PM Hennekens CH Cerkus A Stampfer MJ. Plasma concentrations of cross-linked fibrin degradation product (D-dimer) and the risk of future myocardial infarction among apparently healthy men. Circulation. (1994) 90:2236–40. doi: 10.1161/01.CIR.90.5.2236, PMID: 7955179

[16] Smith FB Lee AJ Fowkes FG Price JF Rumley A Lowe GD. Hemostatic factors as predictors of ischemic heart disease and stroke in the Edinburgh artery study. Artherioscler Thromb Vasc Biol. (1997) 17:3321–5. doi: 10.1161/01.ATV.17.11.3321, PMID: 9409328

[17] Sechi LA Zingaro L Catena C Casaccio D De Marchi S. Relationship of fibrinogen levels and hemostatic abnormalities with organ damage in hypertension. Hypertension. (2000) 36:978–85. doi: 10.1161/01.HYP.36.6.978, PMID: 11116111

[18] Varugese GI Lip GYH. Is hypertension a prothrombotic state? Curr Hypertens Rep. (2005) 7:168–73. doi: 10.1007/s11906-005-0005-4 15913489

[19] Sechi LA Zingaro L Catena C De Marchi S. Increased fibrinogen levels and hemostatic abnormalities in patients with arteriolar nephrosclerosis: association with cardiovascular events. Thromb Haemost. (2000) 84:565–70. doi: 10.1055/s-0037-1614068, PMID: 11057851

[20] Catena C Colussi G Brosolo G Sechi LA. A prothrombotic state is associated with early arterial damage in hypertensive patients. J Artheroscler Thromb. (2012) 19:471–8. doi: 10.5551/jat.10819, PMID: 22659531

[21] Catena C Colussi G Fagotto V Sechi LA. Decreased fibrinolitic activity is associated with carotid artery stiffening in arterial hypertension. J Res Med Sci. (2017) 22:57. doi: 10.4103/jrms.JRMS_619_16, PMID: 28616044 PMC5461593

[22] Catena C Colussi G Fedrizzi S Sechi LA. Association of a prothrombotic state with left-ventricular diastolic dysfunction in hypertension: a tissue-Doppler imaging study. J Hypertens. (2013) 31:2077–84. doi: 10.1097/HJH.0b013e328362d951, PMID: 24107736

[23] Catena C Zingaro L Casaccio D Sechi LA. Abnormalities of coagulation in hypertensive patients with reduced creatinine clearance. Am J Med. (2000) 109:556–61. doi: 10.1016/S0002-9343(00)00567-2, PMID: 11063957

[24] Catena C Colussi G Novello M Fagotto V Sechi LA. Intrarenal vascular resistance is associated with a prothrombotic state in hypertensive patients. Kidney Blood Press Res. (2016) 41:929–36. doi: 10.1159/000452594, PMID: 27894116

[25] Sechi LA Novello M Colussi GL Di Fabio A Chiuch A Nadalini E. Relationship of plasma renin with a prothrombotic state in hypertension: relevance for organ damage. Am J Hypertens. (2008) 21:1347–53. doi: 10.1038/ajh.2008.293, PMID: 18948960

[26] Tay K-H Lip GYH. What drives the link between the renin-angiotensin-aldosterone system and the prothrombotic state in hypertension? Am J Hypertens. (2008) 21:1278–9. doi: 10.1038/ajh.2008.315, PMID: 19020509

[27] Kwaan HC. Role of plasma proteins in whole blood viscosity: a brief clionical review. Clin Hemorheol Microcirc. (2010) 44:167–76. doi: 10.3233/CH-2010-1271, PMID: 20364062

[28] Sie MP Isaac A de Maat MP Mattace-Raso FU Uitterlinden AG Kardys I. Genetic variation in the fibrinogen-alpha and fibrinogen-gamma genes in relation to arterial stiffness: the Rotterdam study. J Hypertens. (2009) 27:1392–8. doi: 10.1097/HJH.0b013e32832a95b0, PMID: 19412134

[29] Lieb W Larson MG Benjamin EJ Yin X Tofler GH Selhub J. Multimarker approach to evaluate correlates of vascular stiffness: the Framingham heart study. Circulation. (2009) 119:37–43. doi: 10.1161/CIRCULATIONAHA.108.816108, PMID: 19103986 PMC2722113

[30] McEniery CM Spratt M Munnery M Yarnell J Lowe GD Rumlry A. An analysis of prospective risk factors for aortic stiffness in men: 20-year follow-up from the Caerphilly prospective study. Hypertension. (2010) 56:36–43. doi: 10.1161/HYPERTENSIONAHA.110.150896 20530296

[31] Wykretowicz J Guzik P Krauze T Marciniak R Komarnicki M Piskorski J. Fibrinogen and D-dimer in contrasting relation with measures of wave reflection and arterial stiffness. Scand J Clin Lab Invest. (2012) 72:629–34. doi: 10.3109/00365513.2012.727023, PMID: 23020230

[32] Vlachopoulos C Pietri P Aznaoridis K Vyssoulis G Vasiliadou C Bratsas A. Relationship of fibrinogen with arterial stiffness and wave reflections. J Hypertens. (2007) 25:2110–6. doi: 10.1097/HJH.0b013e3282dc25da, PMID: 17885555

[33] Nakashima MO Rogers HJ. Hypercoagulable states: an algorithmic approach to laboratory testing and update on monitoring of direct oral anticoagulants. Blood Res. (2014) 49:85–94. doi: 10.5045/br.2014.49.2.85, PMID: 25025009 PMC4090343

[34] Catena C Colussi GL Capobianco F Brosolo G Sechi LA. Uricaemia and left ventricular mass in hypertensive patients. Eur J Clin Investig. (2014) 44:972–81. doi: 10.1111/eci.12331, PMID: 25186106

[35] Williams B Mancia G Spiering W Agabiti Rosei E Azizi M Burnier M. 2018 ESC/ESH guidelines for the management of arterial hypertension. J Hypertens. (2018) 36:1953–2041. doi: 10.1097/HJH.0000000000001940 30234752

[36] Brosolo G Catena C Da Porto A Bulfone L Vacca A Verheyen ND. Differences in regulation of cortisol secretion contribute to left ventricular abnormalities in patients with essential hypertension. Hypertension. (2022) 79:1435–44. doi: 10.1161/HYPERTENSIONAHA.122.19472, PMID: 35535606

[37] Brosolo G Da Porto A Bulfone L Scandolin L Vacca A Bertin N. Vitamin D deficiency is associated with glycometabolic changes in nondiabetic patients with arterial hypertension. Nutrients. (2022) 14:311. doi: 10.3390/nu14020311, PMID: 35057492 PMC8778458

[38] Catena C Brosolo G Da Porto A Donnini D Bulfone L Vacca A. Association of non-alcoholic fatty liver disease with left ventricular changes in treatment-naïve patients with uncomplicated hypertension. Front Cardiovasc Med. (2022) 9:1030968. doi: 10.3389/fcvm.2022.1030968, PMID: 36312275 PMC9606246

[39] Catena C Colussi GL Brosolo G Verheyen N Novello M Bertin N. Long-term renal and cardiac outcomes after stenting in patients with resistant hypertension and atherosclerotic renal artery stenosis. Kidney Blood Press Res. (2018) 42:774–83. doi: 10.1159/000484299 29161704

[40] Sechi LA Catena C Casaccio D Zingaro L. Lipoprotein(a), haemostativc variables and cardiovascular damage in hypertensive patients. J Hypertens. (2000) 18:709–16. doi: 10.1097/00004872-200018060-00008, PMID: 10872555

[41] Nichol WW Singh BM. Augmentation indexes a measure of peripheral vascular disease state. Curr Opin Cardiol. (2002) 17:543–51. doi: 10.1097/00001573-200209000-00016 12357133

[42] vanLeeuwen-Segarceanu EM Tromp W Bos WJW Vogels O Groothoff JW van de Lee JH. Comparing two instruments measuring carotid-femoral pulse wave velocity: Vicorder versus Sphygmocor. J Hypertens. (2010) 28:1687–91. doi: 10.1097/HJH.0b013e32833a8b83, PMID: 20498619

[43] Benetos A Laurent S Hoeks AP Boutouyrie PH Safar ME. Arterial alterations with ageing and high blood pressure. Arterioscler Thromb. (1993) 13:90–7. doi: 10.1161/01.ATV.13.1.90, PMID: 8422344

[44] Emoto M Nishizawa Y Kawagishi T Maekawa K Hiura Y Kanda H. Stiffness indexes beta of the common carotid and femoral arteries are associated with insulin resistance in NIDDM. Diabetes Care. (1998) 21:1178–82. doi: 10.2337/diacare.21.7.1178, PMID: 9653616

[45] Safar ME Laurent S Pannier BM London GM. Structural and functional modifications of peripheral large arteries in hypertensive patients. J Clin Hypertens. (1987) 3:360–7. PMID: 2889808

[46] Bots ML Dijk JM Oren A Grobbee DE. Carotid intima-media thickness, arterial stiffness and risk of cardiovascular disease: current evidence. J Hypertens. (2002) 20:2317–25. doi: 10.1097/00004872-200212000-00002 12473847

[47] Townsend RR Wilkinson IB Schiffrin EL Avolio AP Chirinos JA Cockroft JR. Recommendations for improving and standardizing vascular research on arterial stiffness: a scientific statment from the American Heart Association. Hypertension. (2015) 66:698–722. doi: 10.1161/HYP.0000000000000033, PMID: 26160955 PMC4587661

[48] Lip GY Blann AD. Does hypertension confer a hypercoagulable state? Vircow’s triad revisited. Circulation. (2000) 101:218–20. doi: 10.1161/01.CIR.101.3.218, PMID: 10645912

[49] Poli KA Tofler GH Larson MG Evans JC Sutherland PA Lipinska I. Association of blood pressure with fibrinolytic potential in the Framingham offspring population. Circulation. (2000) 101:264–9. doi: 10.1161/01.CIR.101.3.264, PMID: 10645922

[50] Catena C Novello M Lapenna R Baroselli S Colussi GL Nadalini E. New risk factors for atherosclerosis in hypertension: focus on the prothrombotic state and lipoprotein(a). J Hypertens. (2005) 23:1617–31. doi: 10.1097/01.hjh.0000178835.33976.e7, PMID: 16093903

[51] Thompson WD Sabally K Smith EB Benjamin N. Stimulation of proliferation of smooth muscle cell and fibroblasts in culture by fibrin degradation products and human atherosclerotic plaque extracts. Blood Coagul Fibrinolysis. (1994) 5:43–8.7514044

[52] Wilkinson IB MacCxallum H Cockroft JR Webb DJ. Inhibition of basal nitric oxide synthesis increased aortic augmentation index and pulse wave velocity in vivo. Br J Clin Pharmacol. (2022) 53:189–92. doi: 10.1046/j.1365-2125.2002.1528adoc.x PMC1874288 11851643

[53] Taquet A Bonithon-Kopp C Simon A Levenson J Scarabin Y Malmejac A. Relations of cardiovascular risk factors to aortic pulse wave velocity in asymptomatic middle-aged women. Eur J Epidemiol. (1993) 9:298–306. doi: 10.1007/BF00146267, PMID: 8405315

[54] Nishiwaki Y Takebayashi T Omae K Ishizuka C Nomiyama T Sakuray H. Relationship between the blood coagulation-fibrinolysis system and the subclinical indicators of arteriosclerosis in a healthy male population. J Epidemiol. (2000) 10:34–41. doi: 10.2188/jea.10.34, PMID: 10695259

[55] Schnabel R Larson MG Dupuis J Lunetta KL Lipinska I Meigs JB. Relations of inflammatory biomarkers and common genetic variants with arterial stiffness and wave reflection. Hypertension. (2008) 51:1651–7. doi: 10.1161/HYPERTENSIONAHA.107.105668, PMID: 18426996 PMC2892983

[56] Mitu F Mitu O Dimitriu C Dimitriu G Mitu M. Significance of arterial stiffness and relationship with other noninvasive methods for the assessment of subclinical atherosclerosis in patients with metabolic syndrome. Rev Med Chir Soc Med Nat Iasi. (2013) 117:59–64. PMID: 24505893

[57] Kampus P Muda P Kals J Ristimäe T Fischer K Teesalu R. The relationship between inflammation and arterial siffness in patients with essential hypertension. Int J Cardiol. (2006) 112:46–51. doi: 10.1016/j.ijcard.2005.08.026, PMID: 16297996

[58] Huybrechts SAM Devos DG Vermeersch SJ Mahieu D Achten E de Backer TLM. Carotid to femoral pulse wave velocity: a comparison of real travelled aortic path lengths determined by MRI and superficial measurements. J Hypertens. (2011) 29:1577–82. doi: 10.1097/HJH.0b013e3283487841 21666491

[59] Ferreira I Boreham CA Twisk JW Gallagher AM Young IS Murray LJ. Clustering of metabolic syndrome risk factors and arterial stiffness in young adults: the Northern Ireland Young heart project. J Hypertens. (2007) 25:1009–20. doi: 10.1097/HJH.0b013e3280a94e76, PMID: 17414665

